# Phosphorylation of Yun is required for stem cell proliferation and tumorigenesis

**DOI:** 10.1111/cpr.13230

**Published:** 2022-04-18

**Authors:** Xuejing Ren, Hang Zhao, Lin Shi, Zhengran Li, Ruiyan Kong, Rui Ma, Lemei Jia, Shan Lu, Jian‐Hua Wang, Meng‐qiu Dong, Yingchun Wang, Zhouhua Li

**Affiliations:** ^1^ College of Life Sciences Capital Normal University Beijing China; ^2^ Department of Neurology Capital Medical University Beijing China; ^3^ National Institute of Biological Sciences Beijing China; ^4^ State Key Laboratory of Plant Genomics, Institute of Genetics and Developmental Biology, The Innovative Academy of Seed Design Chinese Academy of Sciences Beijing China; ^5^ College of Advanced Agricultural Sciences University of Chinese Academy of Sciences Beijing China

## Abstract

Stem cells maintain adult tissue homeostasis under physiological conditions. Uncontrolled stem cell proliferation will lead to tumorigenesis. How stem cell proliferation is precisely controlled is still not fully understood. Phosphorylation of Yun is essential for ISC proliferation. Yun is essential for the proliferation of normal and transformed intestinal stem cells. Our mass spectrometry and biochemical data suggest that Yun can be phosphorylated at multiple residues in vivo. Interestingly, we show that the phosphorylation among these residues is likely interdependent. Furthermore, phosphorylation of each residue in Yun is important for its function in ISC proliferation regulation. Thus, our study unveils the important role of post‐translational modification of Yun in stem cell proliferation.

## Graphical abstract

Yun (Luck in Chinese) is an intrinsic factor identified for intestinal stem cell (ISC) proliferation control. Yun is post‐translational modified by phosphorylation at five serine residues at its C‐terminus in vivo. Phosphorylation among these five residues is likely interdependent. Importantly, phosphorylation of each residue in Yun is important for Yun's function in ISC proliferation regulation.

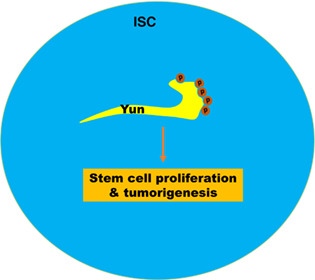

## 1 INTRODUCTION

Tissue homeostasis is maintained by residential stem cells, which proliferate and produce differentiated progeny to replenish lost cells. Thus, the proliferation (self‐renewal) and differentiation of adult stem cells must be tightly balanced under physiological conditions. Disruption of this balance will result in excessive stem cell or precocious stem cell differentiation and finally stem cell depletion, eventually leading to various diseases, such as cancer and precocious aging.^1^, ^2^, ^3^ In particular, it has been proposed that various tumours possess cancer stem cells (CSCs), which are the driving force of tumour development and progression, dormancy, and recurrence.^4^, ^5^, ^6^ Therefore, illustrating the underlying mechanisms of stem cell proliferation control is critical for understanding homeostasis regulation and for the development of potential therapeutics to treat human diseases including cancer.

The adult *Drosophila* intestine is an excellent model to study the regulation of stem cell proliferation/differentiation and tumorigenesis, which shares marked similarities in terms of development, cellular makeup, and genetic control with its mammalian counterparts.^7^, ^8^, ^9^, ^10^, ^11^ *Drosophila* intestinal stem cells (ISCs) are distributed along the basement membrane of the adult intestinal epithelium^12^, ^13^ and divided asymmetrically to produce differentiating enteroblasts (EBs) or EE progenitors (EEPs). One of the Notch ligands, Delta, is specifically expressed in ISCs, while the Notch receptor is expressed in ISCs, EBs, and EEPs (termed progenitors collectively). Notch signalling activation in EBs promotes their differentiation into absorptive enterocytes (ECs).^12^, ^14^, ^15^, ^16^, ^17^ Recent studies show that EE cells may not be generated from EBs, but directly from ISCs or EEPs, which divide once to produce two EEs.^18^, ^19^, ^20^ The proliferation and differentiation of ISCs are regulated by multiple signalling pathways such as the Notch, Wingless (Wg), JAK/STAT, EGFR, Hippo, Insulin, Hedgehog, and BMP signalling to maintain tissue homeostasis under physiological and stressed conditions (see reviews^21^, ^22^, ^23^, ^24^ and references therein).

In our recent study, we identified a novel intrinsic factor Yun (Luck in Chinese) which sustains the proliferation of normal and transformed ISCs.^25^ Yun is a novel protein without any known domains or motifs. During the course of our study, another group showed that it is implicated in cell proliferation in larval brain and spermatogenesis with unknown mechanisms and named it as *diamond* (*dind*).^26^ Yun is required for the proliferation of ISCs under physiological conditions and tissue regeneration under stress conditions.^25^ The identification of Yun as an essential stem cell proliferation regulator has important applications, which can be used as a target to treat intestinal malignancies. However, it remains unexplored whether its activity in ISC proliferation is regulated by any post‐translational modifications (PTMs).

In this study, we investigate whether PTMs exist in Yun and whether these PTMs are required for Yun's function in ISC proliferation. We find that Yun is phosphorylated in vivo. Several phosphorylation residues are identified by mass spectrometry. Site‐directed mutagenesis analyses indicate that the phosphorylation of these residues may be interdependent and is important for the proper function of Yun in ISC proliferation regulation.

## 2 RESULTS

### 2.1 Yun is a positive regulator of ISC proliferation

**FIGURE 1:**
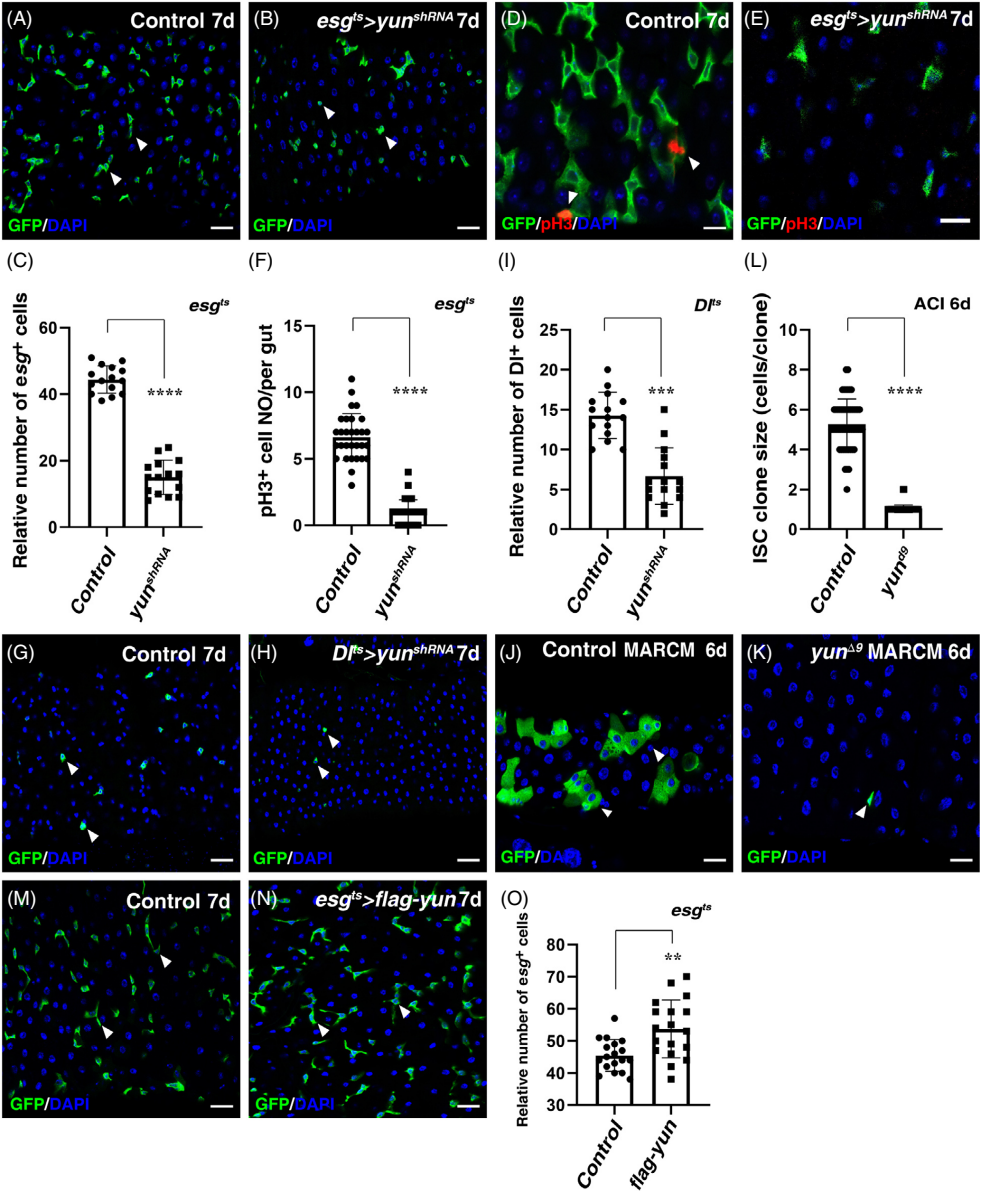
Yun is required for ISC proliferation. (A) *esg* ^+^ cells (green, by *esg* > *GFP*) in Control intestines at 29°C for 7 days (white arrowheads). (B) The number of *esg* + cells (green) is significantly decreased in *esg* ^*ts*^ > *yun* ^*shRNA*^ intestines at 29°C for 7 days compared to control (white arrowheads). (C) Quantification of the relative number of *esg* + cells in Control and *esg* ^*ts*^ > *yun* ^*shRNA*^ intestines. Mean ± SD is shown. n = 15. ****p < 0.0001. (D) pH 3 staining (red) in Control intestines at 29°C for 7 days (white arrowheads). (E) pH 3 staining (red) in *esg* ^*ts*^ > *yun* ^*shRNA*^ intestines at 29°C for 7 days. (F) Quantification of the number of pH 3 cells per gut in Control and *esg* ^*ts*^ > *yun* ^*shRNA*^ intestines. Mean ± SD is shown. *n* = 20. ****p < 0.0001. (G) ISCs (green, by *Dl* > *GFP*) in Control intestines at 29°C for 7 days (white arrowheads). (H) Compared with Control, the number of ISCs (green) is significantly decreased in *Dl* ^*ts*^ > *yun* ^*shRNA*^ intestines at 29°C for 7 days (white arrowheads). (I) Quantification of the relative number of ISCs in Control and *Dl* ^*ts*^ > *yun* ^*shRNA*^ intestines. Mean ± SD is shown. n = 15. ****p < 0.0001. (J) MARCM clones of FRT Control (green) at room temperature for 6 days after clone induction (ACI). (K) The clone size of *yun* ^*Δ9*^ is significantly decreased (ACI 6 days). (L) Quantification of the clone size in Control and *yun* ^*Δ9*^ mutant. Mean ± SD is shown. *n* = 15. ****p < 0.0001. (M) *esg* + cells (green, by *esg* > *GFP*) in Control intestines at 29°C for 7 days (white arrowheads). Note that this panel is same as panel A for easy comparison. (N) The number of *esg* + cells (green) is significantly increased in *esg* ^*ts*^ > *flag‐yun* intestines at 29°C for 7 days compared to control (white arrowheads). (O) Quantification of the relative number of *esg* + cells in Control and *esg* ^*ts*^ > *flag‐yun* intestines. Mean ± SD is shown. *n* = 18. **p < 0.01. In all panels except graphs, GFP is in green and blue indicates DAPI staining of DNA. Scale bars: 20 μm

To identify regulators involved in ISC maintenance and proliferation/differentiation, we carried out a genome‐wide RNAi screen in *Drosophila* adult posterior midgut using an *esgGal4, UAS‐GFP, tubGal80* ^*ts*^ (*esg* ^*ts*^) driver, which is expressed in the progenitors (ISCs, EBs, and EEPs).^25^, ^27^, ^28^ From the screen, we identified many known and novel ISC regulators, including Yun (encoded by *CG7705*).^25^ *Yun* encodes a novel protein without any known domains or motifs. We examined its expression pattern by immunostaining using a Yun‐specific antibody and found that in adult intestines, Yun was mainly expressed in progenitors and EEs (Figure S1). Compared to the control, knocking down *yun* by expressing an effective shRNA RNAi line (*yun* ^*shRNA*^) showed a significant reduction in the number of *esg* ^+^ cells (Figures 1A–C and S2). Consistently, the number of ISCs undergoing mitosis was significantly reduced in *esg* ^*ts*^ > *yun* ^*shRNA*^ intestines (Figures 1D–F). However, the observed ISC proliferation defects in the absence of Yun were not caused by cell death, indicating that Yun is required for the proliferation of normal ISCs under physiological conditions (Figure S3). We further examined whether Yun is required in ISCs for ISC proliferation control by depleting *yun* using an ISC‐specific driver, *Dl‐Gal4*. Depletion of Yun specifically in ISCs also resulted in a significant reduction in the number of ISCs (Figures 1G–I). To further confirm the role of Yun played in ISC proliferation control, we generated *yun* null mutant ISC clones using MARCM clone technique.^25^, ^29^ Compared with the control ISC clones which contained multiple cells, most *yun* null mutant ISC clones failed to proliferate and contained only 1 cell per clone, supporting the notion that Yun sustains ISC proliferation under normal conditions (Figures 1J–L). Conversely, ectopic expression of *yun* resulted in significant increase in progenitors (Figures 1M–O). Altogether, these data indicate that Yun is required for ISC proliferation under physiological conditions.

As Yun is also expressed in EE cells, we further examined what will happen in the absence of *yun* in EE cells. We depleted Yun specifically in EE cells using the EE‐specific driver *prosGal4*. No obvious defects were observed when Yun is depleted in EEs, with no significant change of progenitors and EE cells, indicating that Yun in EE cells is dispensable for ISC proliferation (Figure S4).

### 2.2 Yun is required for tumorigenesis

**FIGURE 2:**
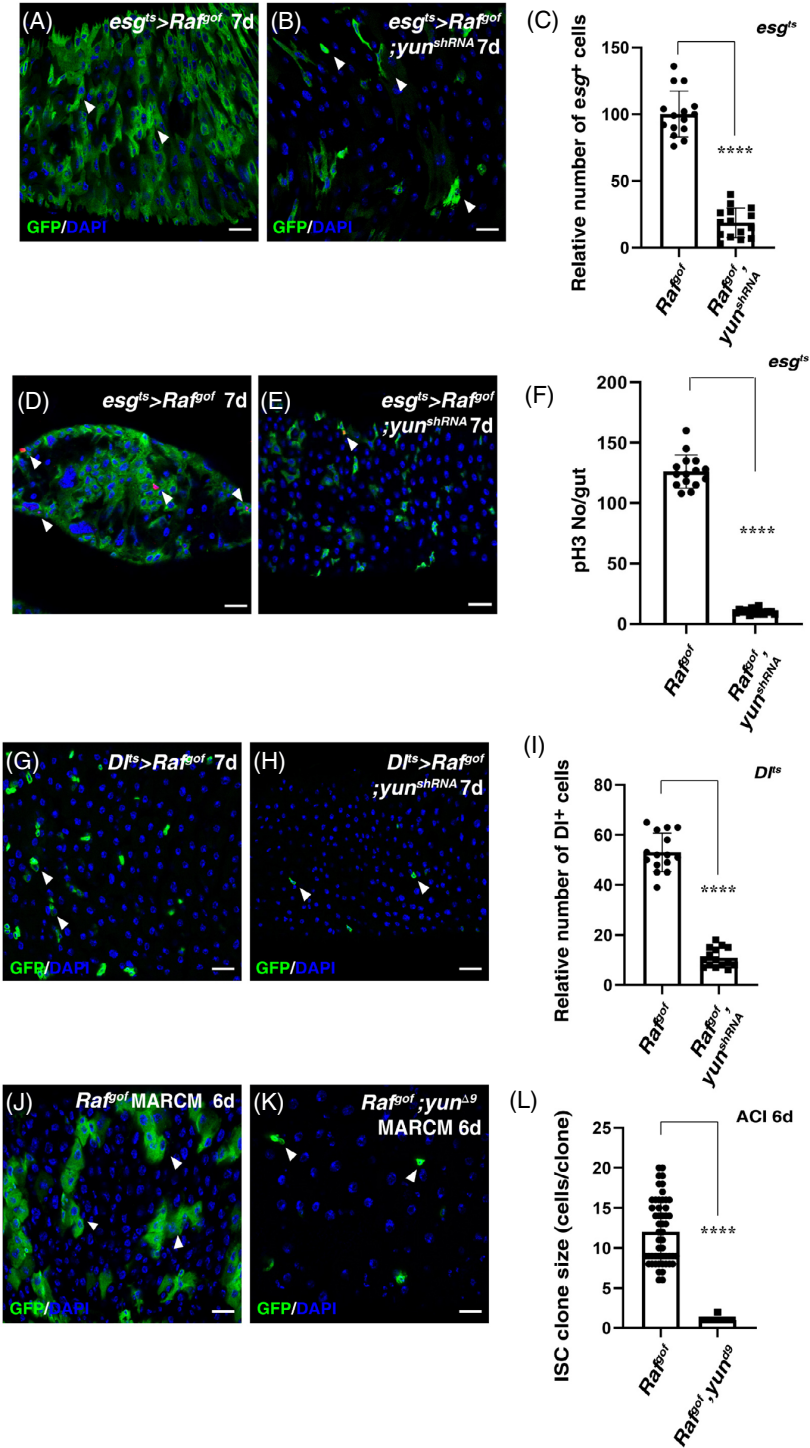
Yun is required for tumorigenesis. (A) Expression of *Raf* ^*gof*^ in *esg* ^+^ cells causes tumours (green, by *esg* > *GFP*) at 29°C for 7 days (white arrowheads). (B) The number of tumour cells in *esg* ^*ts*^ *s Raf* ^*gof*^ intestines (green, by *esg* > *GFP*) is dramatically decreased by co‐expression of *yun* ^*shRNA*^ at 29°C for 7 days (white arrowheads). (C) Quantification of the relative number of ISCs in *esg* ^*ts*^ > *Raf* ^*gof*^ intestines and *esg* ^*ts*^ > *Raf* ^*gof*^ *, yun* ^*shRNA*^ intestines. Mean ± SD is shown. n = 15. ****p < 0.0001. Please note that *esg* ^*ts*^ > *Raf* ^*gof*^ intestines are highly deformed which prevent the accurate quantification of *esg* ^+^ cells in *esg* ^*ts*^ > *Raf* ^*gof*^ intestines. (D) pH 3 (red) in *esg* ^*ts*^ > *Raf* ^*gof*^ intestines at 29°C for 7 days (white arrowheads). (E) pH 3 (red) in *esg* ^*ts*^ > *Raf* ^*gof*^ *, yun* ^*shRNA*^ intestines at 29°C for 7 days (white arrowheads). (F) Quantification of pH 3 number per gut in *esg* ^*ts*^ > *Raf* ^*gof*^ intestines and *esg* ^*ts*^ > *Raf* ^*gof*^ *, yun* ^*shRNA*^ intestines. Mean ± SD is shown. *n* = 15. ****p < 0.0001. (G) Expression of *Raf* ^*gof*^ in ISCs increases ISC number at 29°C for 7 days (white arrowheads). (H) The number of ISCs in *Dl* ^*ts*^ > *Raf* ^*gof*^ intestines is significantly decreased by co‐expression of *yun* ^*shRNA*^ at 29°C for 7 days (white arrowheads). (I) Quantification of the relative number of ISCs in *Dl* ^*ts*^ > *Raf* ^*gof*^ intestines and *Dl* ^*ts*^ > *Raf* ^*gof*^ *, yun* ^*shRNA*^ intestines. Mean ± SD is shown. *n* = 15. ****p < 0.0001. (J) MARCM clones of *Raf* ^*gof*^ (green) at room temperature for 6 days after clone induction (ACI). (K) The size of *Raf* ^*gof*^ clone is totally suppressed in *yun* ^*Δ9*^ (ACI 6 days). (L) Quantification of the clone size of indicated genotypes. Mean ± SD is shown. *n* = 15. ****p < 0.0001. In all panels except graphs, GFP is in green and blue indicates DAPI staining of DNA. Scale bars: 20 μm

We then explored whether *yun* is also required for tumorigenesis. Ectopic expression of a constitutively active Raf (Raf^gof^, an activated component of the EGFR/MAPK signalling pathway) in progenitors causes rapid ISC proliferation and eventually leads to tumorigenesis (Figure 2A).^30^, ^31^ We found that depletion of *yun* effectively suppressed the growth and proliferation of Raf^gof^ tumours (Figures 2A–C), indicating that Yun is essential for tumorigenesis. Consistently, the number of mitotic cells observed in esg^ts^ > *Raf* ^*gof*^ intestines was dramatically suppressed by Yun depletion (Figures 2D–F). We further explored whether Yun specifically functions in ISCs to sustain the growth and proliferation of Raf^gof^ tumour. Ectopic expression of Raf^gof^ in ISCs led to significant increase of *esg* ^+^ cells, forming tumours (Figure 2G). Consistently, depletion of *yun* completely suppressed the proliferation of Raf^gof^ tumours (Figures 2G–I). To further confirm the notion that Yun is required for the proliferation of transformed ISCs derived from Raf^gof^ activation (or cancer stem cells, CSCs), we used MARCM technique to examine the roles of Yun in tumorigenesis. The size of Raf^gof^ MARCM clones is significantly increased compared to that of control, whereas the proliferation of Raf^gof^ MARCM clones was completely suppressed in the absence of *yun* (Figures 2K,L). Collectively, these data demonstrate that Yun is essential for tumorigenesis (and CSC proliferation).

### 2.3 Yun is post‐translationally modified by phosphorylation in vivo

**FIGURE 3:**
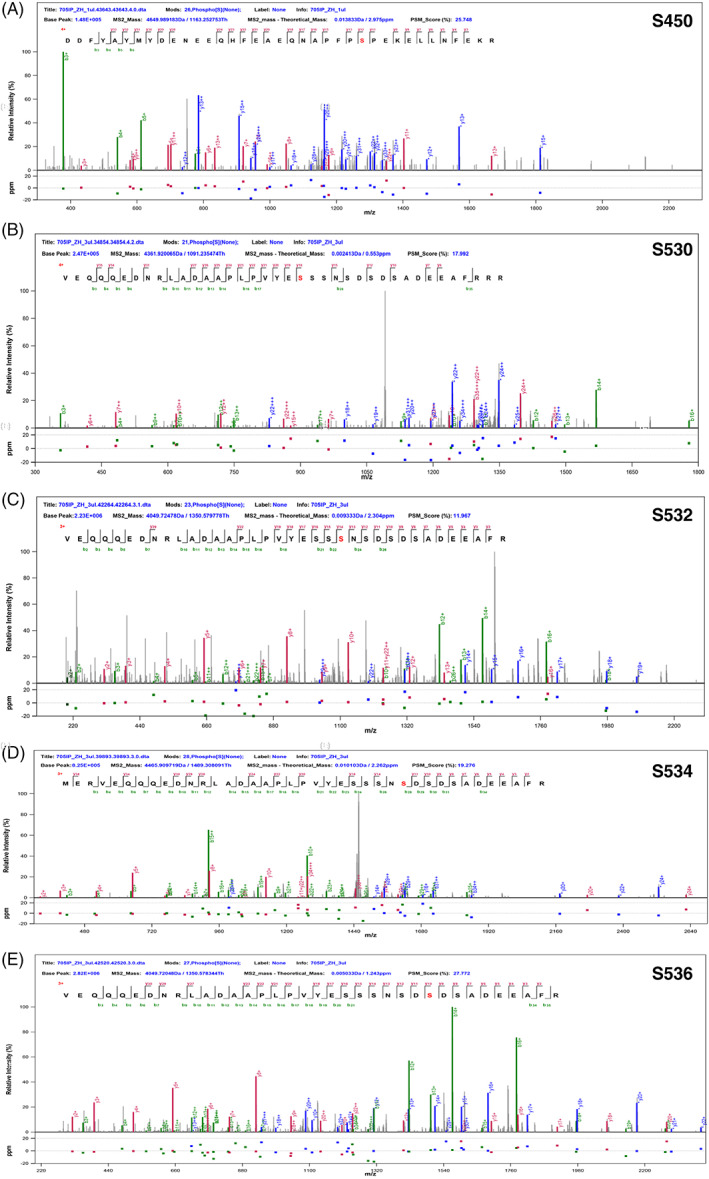
S450, S530, S532, S534, and S536 sites of endogenous Yun protein may be phosphorylated in vivo. (A–E) Five serine residues are likely to be phosphorylated identified by MS analysis in Yun: S450 (A), S530 (B), S532 (C), S534 (D), and S536 (E)

To explore the mechanism of how Yun regulates ISC proliferation and tumorigenesis, we immunoprecipitated endogenous Yun proteins with a 3XFlag tag at its C‐terminus under the control of its endogenous promoter.^25^ Interestingly, double bands of endogenous Yun proteins which were close to each other could be detected in co‐IP experiments, indicating that some kind of post‐translational modification (PTM) may occur on endogenous Yun protein in vivo (Figure S5). We then performed IP‐MS experiments to search for the possible PTM occurred on endogenous Yun protein. Detailed analysis of our mass spectrometry results showed that five serine residues of Yun (S450, S530, S532, S534, and S536) were likely to be phosphorylated (Figure 3). These data indicate that Yun is likely post‐translationally modified by phosphorylation in vivo.

### 2.4 Phosphorylation of different residues of Yun may be interdependent

**FIGURE 4:**
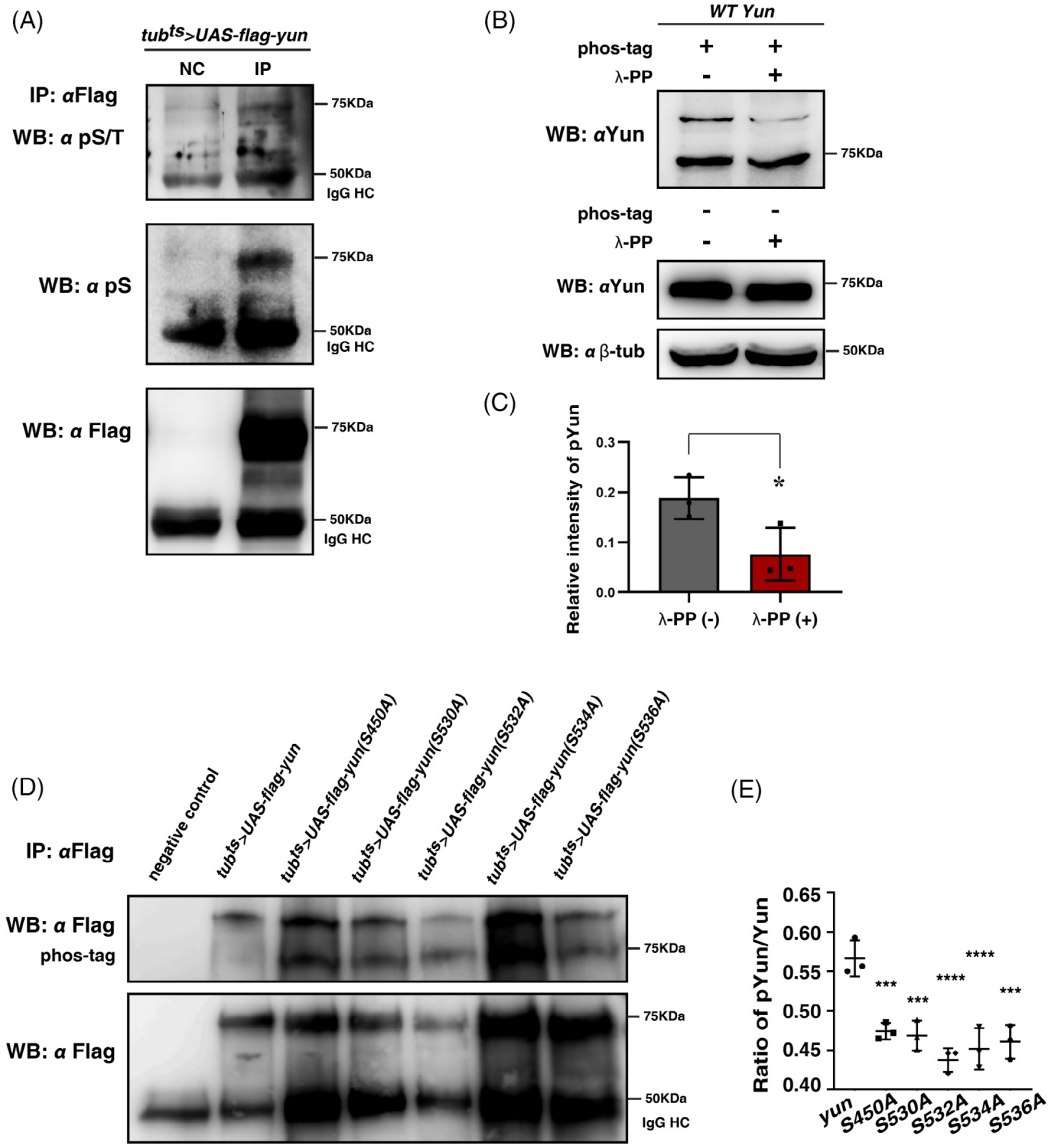
Yun is phosphorylated in vivo. (A) Detection of the phosphorylation status of Yun (pYun) using anti‐phosphorylated serine/threonine (pS/T) and anti‐phosphorylated serine (pS) antibodies by IP and western blot, respectively. NC: negative control. The 50 KDa band is likely the IgG heavy chain (IgG HC). (B) Detection of pYun with phos‐tag and it can be dephosphorylated by λ‐PP. (C) Quantification of the grayscale of pYun with or without λ‐PP. Mean ± SD is shown. *n* = 3. *p < 0.05. (D) Detection of pYun with phos‐tag in flies expressing different Flag‐tagged Yun forms by *tub* ^*ts*^. (E) Quantification of pYun/total Yun ratio of different Yun forms. Mean ± SD is shown. *n* = 3. ***p < 0.001; ****p < 0.0001

To further confirm that Yun protein is phosphorylated in vivo, we pulled down Yun by IP experiments and detected it with phosphorylated serine and/or threonine (pS/T)‐ and phosphorylated serine (pS)‐specific antibodies, respectively. The results showed that Yun could be detected by both antibodies, supporting the notion that Yun is phosphorylated at serine residue(s) in vivo (Figure 4A). Consistently, phosphorylation of Yun could also be detected when analysed with phos‐tag (Figure 4B). Next, we treated the immunoprecipitated Yun proteins with λ‐phosphatase (λ‐PP), which could de‐phosphorylate the phosphorylated proteins. The results showed that treatment with λ‐PP could effectively diminish phosphorylation of Yun proteins (Figure 4B,C). Together, these data show that Yun protein is post‐translationally modified by phosphorylation in vivo.

We further examined the contribution of phosphorylation status of Yun by these candidate phosphorylation residues and whether the phosphorylation status of individual residue can affect phosphorylation of the other residues. We performed site‐directed mutagenesis of these residues, changing each of them to alanine to make phosphorylation dead mutants, and generated transgenic flies carrying these mutants individually (Figure 4D). We then examined the effects of these single sites on the phosphorylation status of Yun using phos‐tag. We found that mutation of each single serine residue significantly affected, but did not completely abolish the phosphorylation of Yun protein, supporting the notion that Yun protein is phosphorylated at multiple serine residues (Figure 4D). Of note, mutation of each single serine residue affected the phosphorylation status of Yun at different extents, implying that phosphorylation of individual residue may affect the phosphorylation status of the other residue(s), thus the phosphorylation of these residues may be interdependent (Figure 4D,E).

### 2.5 Phosphorylation of Yun is critical for ISC proliferation

**FIGURE 5:**
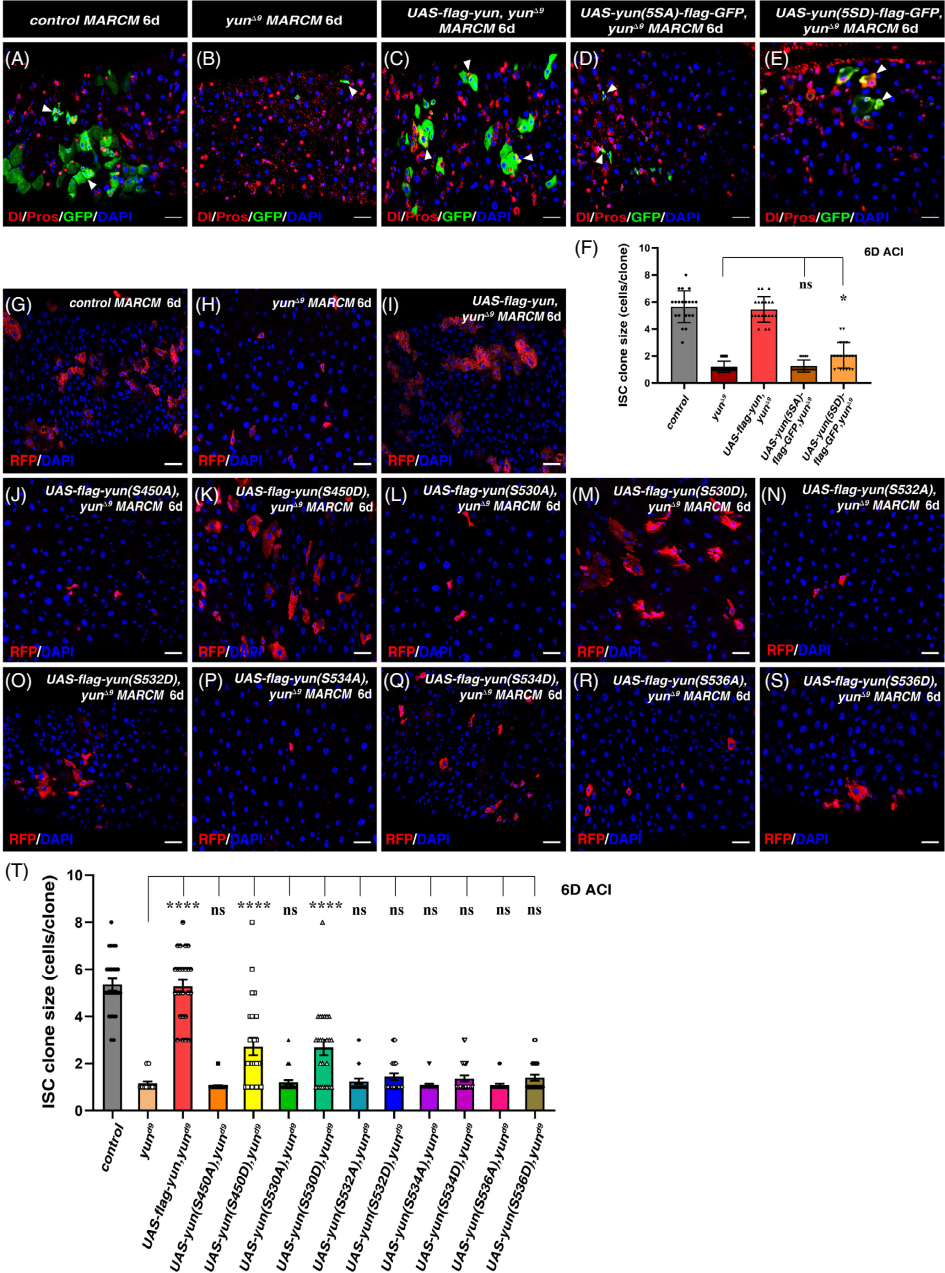
The phosphorylation of the five serine sites in Yun is collectively required for ISC proliferation. (A) MARCM clones of FRT control (green, ACI 6 days), Dl and Pros are in red. (B) The clone size of *yun* ^*Δ9*^ is significantly decreased (green, ACI 6 days). (C) Overexpressing Yun can rescue the clone size of *yun* ^*Δ9*^ (green, ACI 6 days). (D) Expression of *yun(5SA)* mutant cannot rescue the proliferation defects observed in *yun* ^*Δ9*^ (green, ACI 6d). (E) Expression of *yun(5SD)* mutant partially rescues the proliferation defects observed in *yun* ^*Δ9*^ (green, ACI 6 days). (F) Quantification of the clone size of indicated genotypes. Mean ± SD is shown. *n* = 20. ^ns^ *p* >0.05; *p < 0.05. (G) MARCM clones of FRT control (red, ACI 6 days). (H) The clone size of *yun* ^*Δ9*^ is significantly decreased (red, ACI 6 days). (I) Overexpressing *yun* can rescue the clone size of *yun* ^*Δ9*^ (red, ACI 6 days). (J, L, N, P, and R) Expression of different single phospho‐dead point mutants of *yun(1SA)* cannot rescue the proliferation defects observed in *yun* ^*Δ9*^ (red, ACI 6 days). (K, M, O, Q, and S) Expression of different single phospho‐mimic point mutants of *yun(1SD)* partially rescue the proliferation defects observed in *yun* ^*Δ9*^ (red, ACI 6 days). (T) Quantification of the clone size of indicated genotypes. Mean ± SD is shown. n ≥ 20. ^ns^ *p* >0.05; ****p < 0.0001. In all panels except graphs, GFP is in green and blue indicates DAPI staining of DNA. Scale bars: 20 μm

Protein phosphorylation plays an important roles in regulating protein activity and function.^32^, ^33^ To collectively address the role of phosphorylation of these serine residues in ISC proliferation regulation, we constructed two transgenes: one expressed a phosphorylation dead form of all five serine residues (*yun[5SA]*), while the other expressed a phosphorylation mimic form of all five serine residues (*yun[5SD]*), and examined their function. Compared with control MARCM ISC clones, the ISC MARCM clones of *yun* null mutant failed to proliferate (Figure 5A,B). Ectopic expression of wildtype *yun* fully restored the proliferation defects observed in *yun* null mutant (Figures 5A–C and 5F). Whereas ectopic expression of phosphorylation dead *yun(5SA)* failed to restore the proliferation defects observed in *yun* null mutant, suggesting that the phosphorylation of these five serine residues collectively is essential for Yun function in ISC proliferation control (Figure 5D,F). Interestingly, ectopic expression of phosphorylation mimic *yun(5SD)* only partially restored the proliferation defects observed in *yun* null mutant, suggesting that the proper phosphorylation status of each single residue in Yun is also important for its function in ISC proliferation control (Figure 5E,F). Taken together, these data suggest that proper phosphorylation status of these five serine residues collectively is critical for Yun to regulate ISC proliferation.

We then examined the role of phosphorylation of individual serine residue in ISC proliferation regulation in vivo by performing individual rescue experiments using mutated Yun carrying either phosphorylation dead form (SA) or phosphorylation mimic form (SD) of a single residue, respectively. Consistently, ectopic expression of wildtype *yun* could restore the proliferation defects observed in *yun* null mutant (Figures 5G–I and 5T). Interestingly, ectopic expression of phosphorylation dead *yun(S450A)* failed to restore the proliferation defects observed in *yun* null mutant, suggesting that the phosphorylation of this single serine residue is critical for Yun's function in ISC proliferation control (Figure 5J and T). Whereas ectopic expression of phosphorylation mimic *yun(S450D)* significantly restored the proliferation defects observed in *yun* null mutant (Figure 5K and T). These data show that the phosphorylation of the serine 450 residue is important for Yun to regulate ISC proliferation. Similar results were obtained when either *yun(S530A)* or *yun(S530D)* was ectopically expressed, indicating that the phosphorylation of serine 530 residue is also important for Yun's function in ISC proliferation control (Figure 5L, M, and T). Furthermore, ectopic expression of phosphorylation dead *yun(S532A)*, *yun(S534A)*, and *yun(S536A)* failed to restore the proliferation defects observed in *yun* null mutant, while ectopic expression of phosphorylation mimic *yun(S532D)*, *yun(S534D)*, and *yun(S536D)* did not statistically restore the proliferation defects observed in *yun* null mutant, although ISC clones containing 2–3 cells could be occasionally observed, indicating that that the phosphorylation of serine 532, 534, and 536 residues is also necessary for Yun's function in ISC proliferation control (Figures 5N–T). Collectively, these data suggest that phosphorylation of all the five serine residues identified is important for Yun to regulate ISC proliferation, but the role(s) played by individual serine residue may be different and phosphorylation statuses among these residues are likely interdependent.

## 3 DISCUSSION

The proliferation and differentiation of adult stem cells must be tightly balanced in order to maintain tissue homeostasis and prevent tumorigenesis. However, how stem cell proliferation is properly controlled, in particular how post‐translational modifications of intrinsic regulators affect their functions in stem cell proliferation control, remains not fully understood. Here, we find that novel intrinsic Yun is phosphorylated at multiple serine residues in vivo and that the phosphorylation of these serine residues in Yun protein is critical for its function in ISC proliferation control. Interestingly, the phosphorylation of these serine residues may be interdependent and phosphorylation of Yun on the function of Yun in sustaining ISC proliferation seems to be complicated and the role(s) played by individual serine residue may be different.

Protein phosphorylation is one of the most widely observed PTMs, which plays important roles in regulating protein abundance, subcellular localization, function, and activity.^32^, ^33^ Functional analysis of phosphorylated proteins is particularly important for deciphering the causes of diseases like cancer and neurodegenerative diseases, as well as for the development of therapeutic applications.^34^, ^35^ Protein phosphorylation and de‐phosphorylation reactions catalysed by protein kinases and protein phosphatases (PPases) are dynamic which change over time and space. The complex network of protein phosphorylation/de‐phosphorylation provides rapid regulation of the phosphorylated status and supports many fundamental biological functions of cells. Thus identification of the protein kinases and possible PPases of a phosphorylated protein will be critical for unveiling the regulatory network of this protein and elucidating its molecular mechanism. Interestingly, two casein kinases (CKIɑ and CKIIβ) were found to associate with Yun in our IP‐MS results, and their association was further confirmed by co‐IP experiments, implying that they may be the kinases responsible for Yun phosphorylation (Figure S5). Consistent with this possibility, ectopic expression of either CKIɑ or CKIIβ could not restore the proliferation defects observed in the absence of Yun (Figure S6). CKI and CKII are members of Ser/Thr kinases; they interact with many other proteins, such as β‐catenin, other kinases like PIP4K and S6KII, the RNA‐binding protein Orb, circadian proteins(Tim (Timeless) and Per (Period)), and are involved in multiple signalling pathways, including Wnt, Hh, and Hpo.^36^, ^37^, ^38^, ^39^, ^40^, ^41^ The two casein kinases are essential for ISC proliferation. depletion of them compleletly blocks ISC proliferation. Importantly, ectopic expression of both wildtype *yun* and phosphorylation mimic *yun(5SD)* could not restore the proliferation defects in the absence of either casein kinase, suggesting that multiple downstream substrates are required for ISC proliferation control and expression of single substrate is not sufficient to restore ISC proliferation blockage upon their depletion (Figure S5).

Our identification of Yun as a phosphorylated protein provides an interesting cutting point to elucidate the underlying mechanism of how Yun regulates ISC proliferation and tumorigenesis. It will be interesting to systematically investigate how these serine residues of Yun are differentially phosphorylated in ISCs, whether any interdependence(s) of phosphorylation status exists among these residues, and how the phosphorylation of these residues is coordinated to regulate the function of Yun in ISC proliferation control using site‐specific phospho‐Yun antibodies and corresponding knock‐in flies carrying single and/or combined phospho‐dead and phospho‐mimic *yun* mutant in future work.

## 4 EXPERIMENTAL PROCEDURES

### 4.1 Fly lines and husbandry

Flies were maintained on standard media at 25°C. Two–three–days‐old flies were selected and transferred to 29°C, unless otherwise specified. Flies were transferred to new vials with fresh food every day and dissected at specific time points as indicated. In all experiments, only female posterior midguts were analysed. Information about alleles and transgenes used in this study can be found either in FlyBase or as noted: *esgGal4, UAS‐GFP, tubGal80* ^*ts*^ (*esg* ^*ts*^, gift from N. Perrimon), *tubGal80* ^*ts*^ *,tubGal4*, *UAS‐Raf* ^*gof*^ (BL2033), *yun* ^*shRNA*^, *yun* ^*Δ9*^, *UAS‐flag‐yun*, and *hsFlp, ActGal4, UAS‐GFP, FRT82B‐tubGal80* (for MARCM clonal analysis), *hsFlp, ActGal4, UAS‐RFP, FRT82B‐tubGal80* (for MARCM clonal analysis),^25^ *DlGal4, tubGal80* ^*ts*^ (*Dl* ^*ts*^, gifts from S. Hou and R. Xi), *esgGal4, Gal80* ^*ts*^ *, UAS‐CC3Ai*,^42^ *prosGal4, UAS‐GFP, tubGal80* ^*ts*^ (*pros* ^*ts*^, gift from Xiaohang. Yang), *CkIα* ^*v20*^ (THU5314 and HMS02276), *FRT19A‐CkIα* ^*8B12*^ (BL63802), *CkIIβ* ^*v20*^ (THU0596, BL34939, and HMS00058), *FRT19A‐Ck2β* ^*A*^ (BL67713), *UAS‐CkIα‐HA* (BL55067 and BL55068), and *UAS‐CkIIβ* (F001372).

### 4.2 RNAi knockdown and overexpression experiments

Crosses (unless stated otherwise) were maintained at 18°C to bypass potential requirements during early developmental stages. Two–three–days‐old progeny with the desired genotypes were collected after eclosion and maintained at 29°C to inactivate Gal80^ts^ before dissection and immunostaining. The flies were transferred to new vials with fresh food every day.

### 4.3 Constructs and transgenic flies

*pUAST‐flag‐yun* (*CG7705*) was constructed by cloning *yun* ORF (from cDNA clone LP22035, BDGP) into *pUAST‐nflag*. *attB‐pUAST‐CkIIβ‐myc* was constructed by cloning the ORF of *UAS‐CkIIβ* (F001372) into the EcoRI and XbaI site of *attB‐pUAST‐myc*. All point mutation constructs were made by Hieff Mut™ Multi Site‐Directed Mutagenesis Kit (YEASEN, China). *attB‐pUAST‐yun(5SA:S450/530/532/534/536A)‐flag‐GFP* and *attB‐pUAST‐yun(5SD:S450/530/532/534/536D)‐flag‐GFP* vectors were constructed by cloning the mutated *yun(5SA)* and *yun(5SD)* into the EcoRI site of *attB‐pUAST‐flag‐GFP* respectively. *attB‐pUAST‐flag‐yun(1SA:S450A, S530A, S532A, S534A*, or *S536A*, respectively) and *attB‐pUAST‐flag‐yun(1SD:S450D, S530D, S532D, S534D*, or *S536D*, respectively) vectors were constructed by cloning the mutated *yun(1SA)* and *yun(1SD)* into the KpnI site of *attB‐pUAST‐nflag*. Transgenic flies were obtained by standard P‐element‐mediated germline transformation carrying attP site at 36B, 75B, or 86F.

### 4.4 MARCM clone analysis

The clonal analyses were achieved using the MARCM system.^29^ The ISC clones were induced by heat shocking Two–three–days‐old adult flies at 37°C for 60 min. The flies were maintained at a 25°C incubator and transferred to new vials with fresh food every day. The sizes of the marked clones were assayed at 6 days after clone induction (6D ACI, at least 10 midguts for each genotype were assayed).

### 4.5 Immunostainings and fluorescence microscopy

For standard immunostainings, intestines were dissected in 1 × PBS (10 mM NaH_2_PO_4_/Na_2_HPO_4_, 175 mM NaCl, pH 7.4), and fixed in 4% paraformaldehyde for 25 min at room temperature. Samples were washed with 1 x PBT (0.1% Triton X‐100 in 1 × PBS) and blocked in 3% BSA in 1 × PBT for 45 min. Primary antibodies were added to the samples and incubated at 4°C overnight. The following primary antibodies were used: mouse mAb anti‐Dl (C594.9B, 1:50, developed by S. Artavanis‐Tsakonas, DSHB), mouse mAb anti‐Prospero (MR1A, 1:100, developed by Chris Doe, DSHB), rabbit anti‐pH 3 (1:2000, Millipore), rabbit anti‐active caspase 3 (1:1000, Cell Signalling), and rabbit anti‐Yun (1:1000).^25^ Primary antibodies were detected by fluorescent‐conjugated secondary antibodies from Jackson ImmunoResearch Laboratories. Secondary antibodies were incubated for 2 h at room temperature. DAPI (Sigma‐Aldrich; 0.1 μg/ml) was added after secondary antibody staining. The samples were mounted in mounting medium (70% glycerol containing 2.5% DABCO). All images were captured using a Zeiss inverted confocal microscope and were processed in Adobe Photoshop and Illustrator.

### 4.6 Co‐immunoprecipitation and western blotting

Fly tissues were lysed in RIPA buffer [50 mM Tris.HCl, pH 8.0, 150 mM NaCl, 5 mM EDTA, pH 8.0, 0.5% Triton X‐100, 0.5% NP‐40, 0.5% sodium deoxycholate, and complete protease inhibitor cocktail tablets (Roche)] on ice for 30 min. After centrifugation, lysates were then diluted tenfold with RIPA buffer and subjected to immunoprecipitation using anti‐FLAG M2 affinity gel (A2220; Sigma‐Aldrich, USA). The immunocomplexes were collected by centrifugation and washed with 1 ml of RIPA buffer three times. For western blotting, immunoprecipitated proteins were separated in SDS‐PAGE and then blotted onto PVDF membranes. The membranes were stained with primary antibody overnight at 4°C. Followed by washing, PVDF membranes were incubated with secondary antibodies conjugated with HRP, then the membranes were scanned using Luminescent Image Analyser (GE, Sweden). Rabbit anti‐Yun (1:1000),^25^ mouse anti‐Flag (1:1000, Sigma‐Aldrich, USA), Rabbit anti‐pS (1:1000, Abcam), Rabbit anti‐pS/T (1:1000, Abcam), and mouse monoclonal anti‐βTubulin (1:1000, 3G6, Abbkine, USA) antibodies were used. Phos‐tag (Wako, Japan) was added into SDS‐PAGE gel to exhibit phosphorylated Yun. λ‐phosphatase (NEB) was used to dephosphorylate pYun.

### 4.7 MS sample preparation

Proteins were precipitated with 25% trichloroacetic acid (TCA) for at least 30 min on ice. The protein pellets were washed twice with 500 μl ice‐cold acetone, air dried, and then resuspended in 8 M urea, 100 mM Tris, and pH 8.5. After reduction (5 mM TCEP, RT, 20 min) and alkylation (10 mM iodoacetamide, RT, 15 min in the dark), the samples were diluted to 2 M urea with 100 mM Tris, pH 8.5 and digested with trypsin at 1/50 (w/w) enzyme/substrate ratio at 37°C for 16–18 h. The digestion was then stopped by addition of formic acid to 5% (final concentration).

### 4.8 LC–MS/MS analysis

All the samples were analysed using an EASY‐nLC 1000 system (Thermo Fisher Scientific) interfaced with a Q‐Exactive mass spectrometer (Thermo Fisher Scientific). Peptides were loaded on a trap column (75 μm ID, 4 cm long, packed with ODS‐AQ 12 nm‐10 μm beads) and separated on an analytical column (75 μm ID, 12 cm long, packed with Luna C18 1.9 μm 100 Å resin) with a 60 min linear gradient at a flow rate of 200 μl/min as follows: 0–5% B in 2 min, 5–30% B in 43 min, 30–80% B in 5 min, and 80% B for 10 min (A = 0.1% FA, B = 100% ACN, 0.1% FA). Spectra were acquired in data‐dependent mode: the top ten most intense precursor ions from each full scan (resolution 70,000) were isolated for HCD MS2 (resolution 17,500; NCE 27) with a dynamic exclusion time of 30 s. The AGC targets for the MS1 and MS2 scans were 3e6 and 1e5, respectively, and the maximum injection times for MS1 and MS2 were both 60 ms. Precursors with 1+, more than 7+ or unassigned charge states were excluded.

### 4.9 Database search

The MS data were searched against a Uniprot *Drosophila melanogaster* protein database (database ID number of UP000000803) using ProLuCID with the following parameters: precursor mass tolerance, 3 Da; fragment mass tolerance 20 ppm; peptide length, minimum 6 amino acids and maximum 100 amino acids; enzyme, Trypsin, with up to three missed cleavage sites.^43^ The results were filtered by DTASelect requiring FDR <1% at the peptide level and spectra count≥2.^44^ The proteins identified from the negative control and Flag‐Yun IP were contrasted by Contrast.^44^

### 4.10 Data analysis

The number of intestines scored is indicated in the text. To determine the relative number of *esg* ^+^ cells, confocal images of 40× lens/1.0 zoom from a defined posterior midgut region of different genotypes indicated were acquired. The number of *esg* ^+^ cells from each confocal image was determined using Image‐Pro Plus software, manually selecting the “filter” depending on the respective cell size to filter out background signals (referred to as the relative number of *esg* ^+^ cells). The clone sizes were scored manually under Zeiss Imager Z2/LSM780 microscope for indicated genotypes. At least 10 different guts were analysed for each set. Statistical analysis was done using the Student's *t*‐test. PEMS 3.1 software was used for SEM analyses and Sigma plot and GraphPad prism software for graph generation. The graphs were further modified using Adobe Photoshop and Illustrator. ^ns^ *p* >0.05; *p < 0.05; **p < 0.01; *** *p* < 0.001; and **** *p* < 0.0001.

## CONFLICT OF INTEREST

The authors declare no conflicts of interest.

## AUTHOR CONTRIBUTIONS

Conceptualization, Zhouhua Li and Hang Zhao; investigation, Xuejing Ren, Hang Zhao, Lin Shi, Zhengran Li, Ruiyan Kong, Rui Ma, Lemei Jia, Shan Lu, Jian‐Hua Wang, Meng‐qiuDong, Yingchun Wang, Zhouhua Li; formal analysis, Xuejing Ren, Zhouhua Li, Hang Zhao; methodology, Rui Ma; validation, Xuejing Ren, Hang Zhao, and Zhouhua Li; writing—original draft preparation, Xuejing Ren, Hang Zhao, and Zhouhua Li; writing—review and editing, Hang Zhao and Zhouhua Li; supervision, Zhouhua Li; project administration, Zhouhua Li; funding acquisition, Zhouhua Li. All authors have read and agreed to the published version of the manuscript.

## Supporting information


**Appendix S1**Supporting Information

## Data Availability

The data that supports the findings of this study are available in the supplementary material of this article.
