# Supplementary material for: Phosphorylation of Yun is required for stem cell proliferation and tumorigenesis
Source: Cell Prolif. 2022 Apr 18;55(5):e13230. doi: 10.1111/cpr.13230 (PMC9136491; doi:10.1111/cpr.13230)
Supplement: Supplementary file 1 — Appendix S1Supporting Information [file CPR-55-e13230-s001.docx]

**Supplementary Material**

**Phosphorylation of Yun is required for stem cell proliferation and tumorigenesis**

Xuejing Ren, Hang Zhao, Lin Shi, Zhengran Li, Ruiyan Kong, Rui Ma, Lemei Jia, Shan Lu, Jian-Hua Wang, Meng-qiu Dong, Yingchun Wang, and Zhouhua Li

**Supplementary Figures**

**
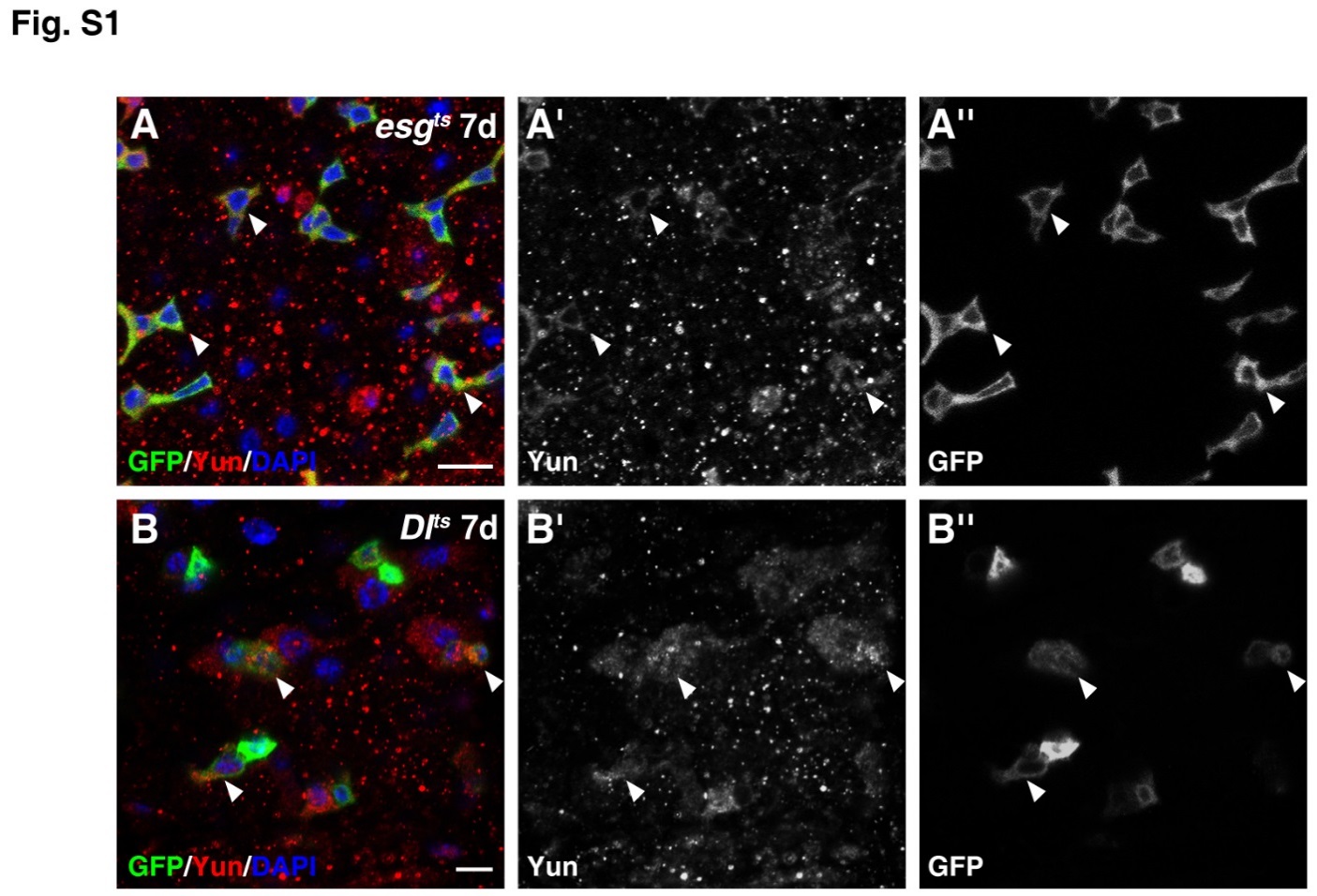
**

**Fig. S1. Yun is mainly expressed in progenitors and EEs in adult midgut.**

(A) Yun (red) is mainly expressed in progenitors (green, by *esg>GFP*) and EEs (the *esg>GFP^-^* diploid cells) in adult midgut (white arrowheads). Yun and GFP channels are showed separately in black white.

(B) Yun (red) is mainly expressed in progenitors (ISCs and EBs, ISCs are in green by *Dl>GFP*) and EEs (the diploid cells away from *Dl>GFP^-^* cells) in adult midgut (white arrowheads). Yun and GFP channels are showed separately in black white.

GFP is in green and blue indicates DAPI staining of DNA. Scale bars: 10 μm.

**
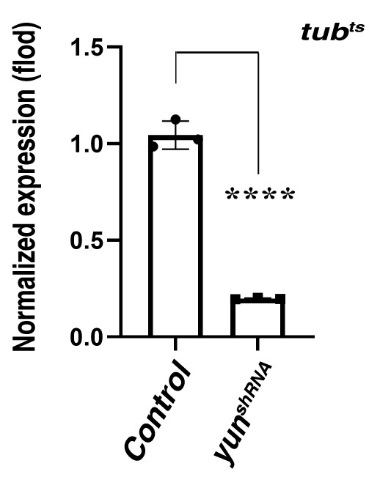
**

**Fig. S2. Knockdown efficacy of *yun^shRNA^*.**

The levels of *yun* transcripts were significantly reduced when *yun* was systematically depleted by *yun^shRNA^*. Mean ± SD is shown. *****p* <0.0001.


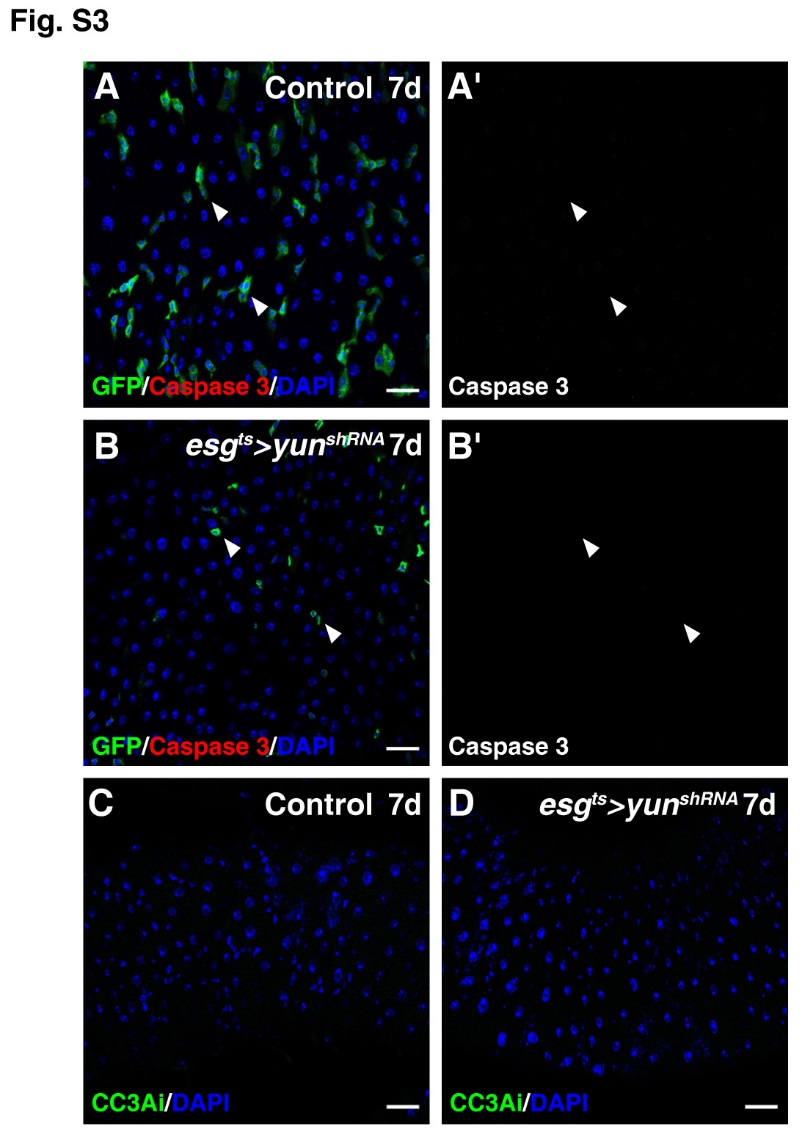


**Fig. S3. Yun affects progenitor proliferation, but not apoptosis.**

(A) Apoptosis of progenitors (red, by active Caspase 3) is barely detectable in Control intestines at 29°C for 7 days (white arrowheads).

(B) No increase of apoptosis is observed in *esg^ts^>yun^shRNA^* intestines at 29°C for 7 days (white arrowheads).

(C) No progenitors undergo apoptosis (green, by CC3Ai) in Control intestines at 29°C for 7 days (white arrowheads).

(D) No progenitors undergo apoptosis (green, by CC3Ai) in *esg^ts^>yun^shRNA^* intestines at 29°C for 7 days (white arrowheads).

GFP is in green and blue indicates DAPI staining of DNA. Scale bars: 20 μm.


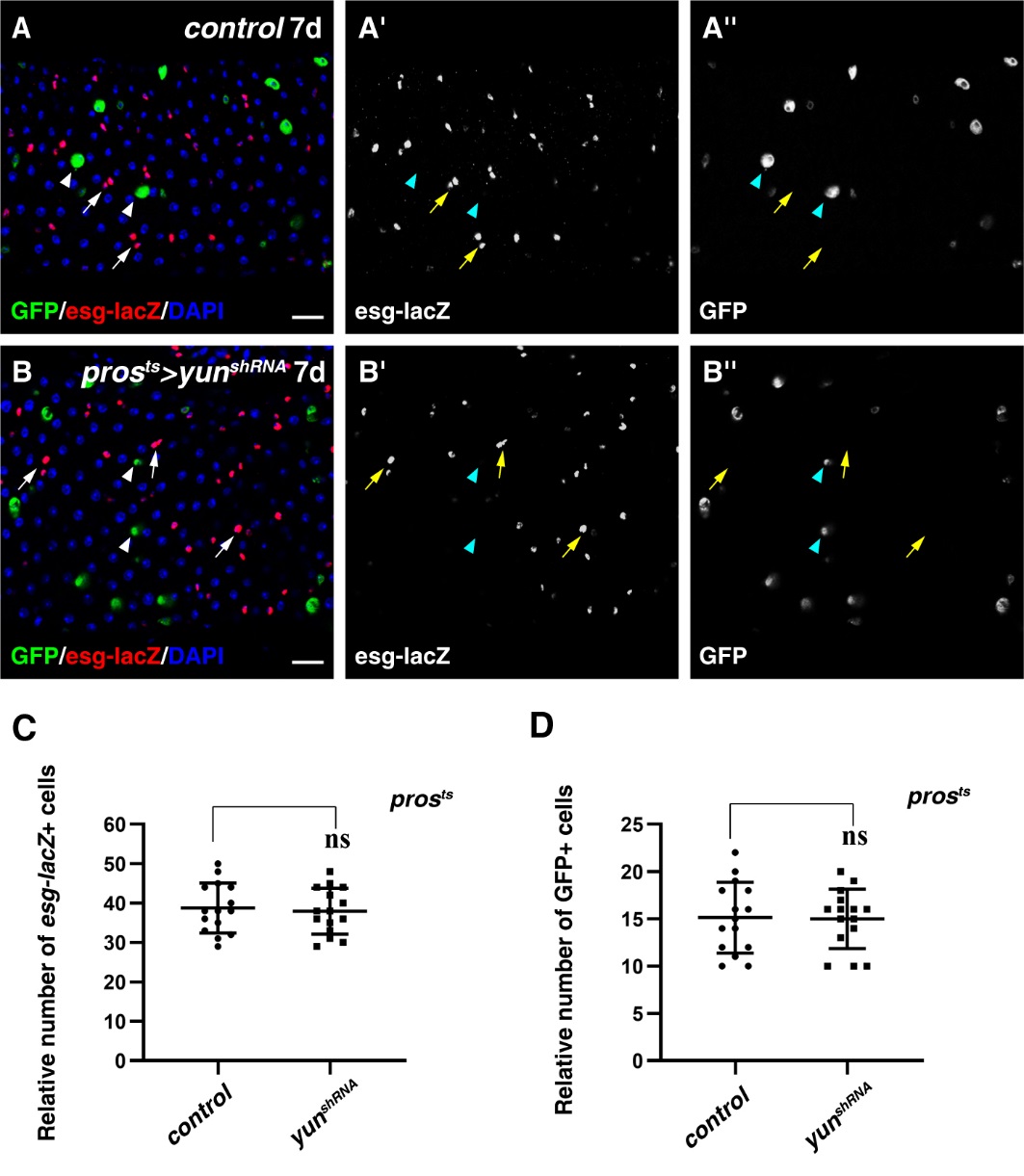


**Fig. S4 Yun is dispensable in EEs for ISC proliferation.**

(A) Progenitors (red, *esg-lacZ*) in control intestines at 29°C for 7 days (white arrowheads). esg-lacZ and GFP channels are showed separately in black white (yellow and cyan arrowheads respectively).

(B) Progenitors (red, *esg-lacZ*) in *pros^ts^>yun^shRNA^* at 29°C for 7 days (white arrowheads).

(C) Quantification of the relative number of progenitors in intestines with indicated genotypes. Mean ± SD is shown. n≥12. ^ns^*P* >0.05.

(D) Quantification of the relative number of EE cells in intestines with indicated genotypes. Mean ± SD is shown. n≥12. ^ns^*P* >0.05.

In all panels except graphs, GFP is in green and blue indicates DAPI staining for DNA. Scale bars: 20 μm.

**
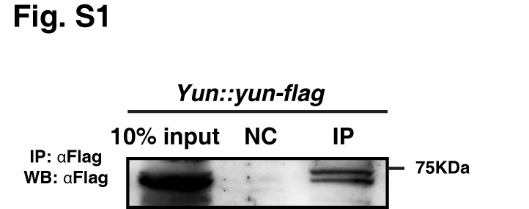
**

**Fig. S5. Two bands of Yun can be detected in IP-WB experiments.**

Two bands of Yun can be detected when endogenous Yun-Flag was isolated by flag-IP and WB. NC: negative control, the same as follows.


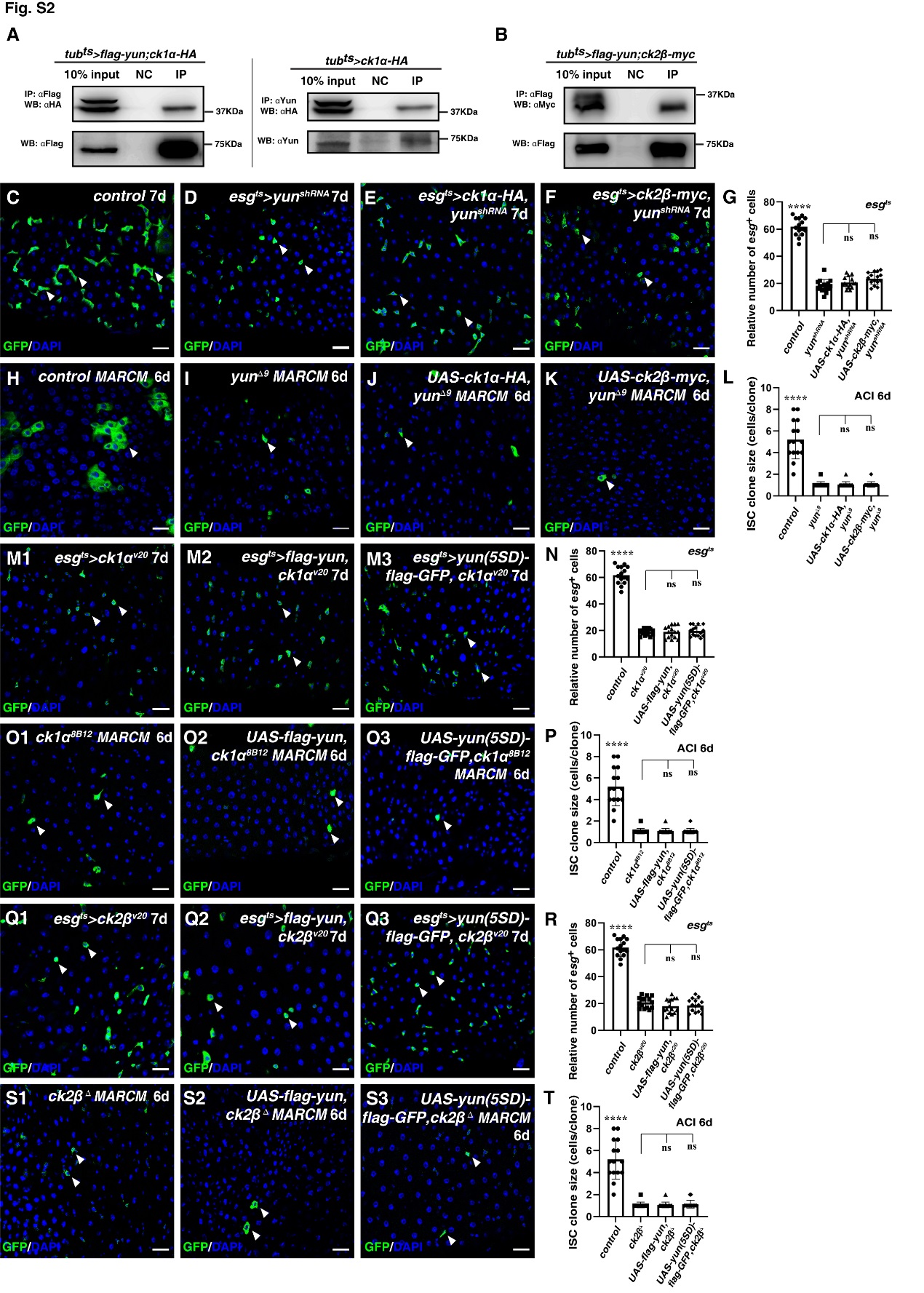


**Fig. S6. Yun may associate with two kinases CKIα and CKIIβ.**

(A) Yun and CKIα mutually interact with each other *in vivo* when overexpressed.

(B) Yun interacts with CK2β *in vivo* when overexpressed.

(C) *esg*^+^ cells (green, by *esg>GFP*) in control intestines at 29°C for 7 days (white arrowheads).

(D) Compared with control, the number of *esg^+^* cells (green) is significantly decreased in *esg^ts^>yun^shRNA^* intestines at 29°C for 7 days (white arrowheads).

(E and F) Overexpression of either CK1α (E) or CK2β (F) cannot rescue the proliferation defects observed in *esg^ts^>yun^shRNA^* intestines at 29°C for 7 days (white arrowheads).

(G) Quantification of the relative number of *esg*^+^ cells in intestines of indicated genotypes. Mean ± SD is shown. n=15. ^ns^*p* >0.05; *****p* <0.0001.

(H) MARCM ISC clones in *FRT* control (green, ACI 6 d).

(I) *yun^Δ9^* ISC MARCMC clones failed to proliferate (green, ACI 6 d).

(J and K) Overexpression of either CK1α (J) or CK2β (K) cannot rescue the proliferation defects observed in *yun^Δ9^* (green, ACI 6 d) (white arrowheads).

(L) Quantification of the ISC clone size in intestines of indicated genotypes. Mean ± SD is shown. n=15. ^ns^*p* >0.05; *****p* <0.0001.

(M) Overexpression of either wildtype *yun* or phosphorylation mimic form of *yun(5SD)* cannot rescue the proliferation defects observed in *esg^ts^>ck1α^v20^* intestines at 29°C for 7 days (white arrowheads).

(N) Quantification of the relative number of *esg^+^* cells in intestines of indicated genotypes. Mean ± SD is shown. n=15. ^ns^*p* >0.05; *****p* <0.0001.

(O) Overexpression of either wildtype *yun* or phosphorylation mimic form of *yun(5SD)* cannot rescue the proliferation defects observed in *ck1α^8B12^* mutant (green, ACI 6d) (white arrowheads).

(P) Quantification of the ISC clone size in intestines of indicated genotypes. Mean ± SD is shown. n=15. ^ns^*p* >0.05; *****p* <0.0001.

(Q) Overexpression of either wildtype *yun* or phosphorylation mimic form of *yun(5SD)* cannot rescue the proliferation defects observed in *esg^ts^>ck2β^v20^* intestines at 29°C for 7 days (white arrowheads).

(R) Quantification of the relative number of *esg*^+^ cells in intestines of indicated genotypes. Mean ± SD is shown. n=15. ^ns^*p* >0.05; *****p* <0.0001.

(S) Overexpression of either wildtype *yun* or phosphorylation mimic form of *yun(5SD)* cannot rescue the proliferation defects observed in *ck2β^A^* mutant (green, ACI 6 d) (white arrowheads).

(T) Quantification of the clone size in intestines of indicated genotypes. Mean ± SD is shown. n=15. ^ns^*p* >0.05; *****p* <0.0001.

In all panels except graphs, GFP is in green and blue indicates DAPI staining of DNA. Scale bars: 20 μm.

**Supplementary Methods**

**qRT-PCR**

RNA was extracted from 30 flies or guts using TRIzol (Invitrogen). RNA was cleaned using RNAeasy (QIAGEN), and complementary DNA (cDNA) was synthesized using the iScript cDNA synthesis kit (Bio-Rad). Quantitative PCR was performed using the iScript one step RT-PCR SYBR green kit (Bio-Rad). Data were acquired using an iQ5 System (Bio-Rad). qRT-PCR was performed in duplicate on each of three independent biological replicates. All results are presented as mean ± SD of the biological replicates. The ribosomal gene *RpL11* was used as the normalization control.

**qRT-PCR primers used:**

*yun*-F: CTGCGGGAGTGCGAGTACTTC

*yun*-R: CAGATCCTCGCTCAGCTGAG

*RpL11*-F: GGTCCGTTCGTTCGGTATTCGC

*RpL11*-R: GGATCGTACTTGATGCCCAGATCG
